# Incidence of Adverse Cardiac Events After Liver Transplantation in Patients With Different Hemodynamic Classifications of Pulmonary Hypertension

**DOI:** 10.1155/ijh/2869285

**Published:** 2026-06-27

**Authors:** David W. Wang, Akira Katayama, Marianne M. Ligon, Samuel E. Crowley, Abhijeet M. Sathe, Ezeldeen Abuelkasem

**Affiliations:** ^1^ Department of Anesthesiology and Perioperative Medicine, University of Pittsburgh School of Medicine, Pittsburgh, Pennsylvania, USA, pitt.edu

## Abstract

**Introduction:**

Pulmonary hypertension (PH) in liver transplantation candidates is associated with elevated morbidity and mortality. Few studies have investigated whether different categories of PH based off hemodynamic characteristics portend worse outcomes. Furthermore, there have been newly defined parameters for PH classifications. Therefore, we aimed to compare postoperative outcomes after liver transplantation in patients within different newly established hemodynamic categories of PH.

**Methods:**

This was a 20‐year single‐center retrospective observational study of adult patients undergoing liver transplantation. Patients with a diagnosis of PH and confirmatory right heart catheterization (RHC) were included in the study. Based off pulmonary artery wedge pressure (PAWP) and Wood units (WU), patients were categorized into four groups: precapillary (PrePH), isolated postcapillary (IpcPH), combined postcapillary (CpcPH), unclassified (UncPH). Our primary outcome was a composite of adverse cardiac events within 30 days postoperatively. Secondary outcomes included prolonged ventilation, acute kidney injury, and mortality at 30 days, 6 months, and 1 year.

**Results:**

Of the 2409 patients who underwent liver transplantation in the study period, 80 patients met criteria for inclusion. The incidences of ACEs were 0% in PrePH, 31.4% in IpcPH, 25.0% in CpcPH, and 10.3% in UncPH (*p* = 0.04). Patients with postoperative ACEs had elevated PAWP (18.1 vs. 14.0 mmHg, *p* = 0.01) and lower pulmonary vascular resistance (0.79 vs 1.3 WU, *p* = 0.01). There were no differences observed in secondary outcomes.

**Conclusion:**

In this single‐center study, we observed that patients with isolated precapillary PH did not have adverse cardiac events. Instead, patients with elevated PAWP, indicating postcapillary PH, were more likely associated with adverse cardiac events. This observation is seen despite milder pulmonary disease, as intended by newly established definitions for PH. Further investigation is warranted, as there may be prognostic implications that can drive future guidelines for liver transplantation in patients with PH.

## 1. Introduction

Portopulmonary hypertension (PoPH) is a serious pulmonary vascular complication that arises in the setting of portal hypertension, most commonly due to liver cirrhosis [1, 2]. Although PoPH is relatively uncommon, it significantly increases perioperative risk and is associated with poor posttransplant outcomes [3, 4]. Current clinical practice permits liver transplantation (LT) in patients with mild PoPH; however, those with moderate to severe PoPH are considered contraindicated for LT unless hemodynamics can be optimized with pulmonary vasodilator therapy [5]. Although PoPH has received increasing attention in the context of cirrhosis, it is not the only cause of pulmonary hypertension (PH) in patients with end‐stage liver disease (ESLD).

In general, PH can be classified into four hemodynamic phenotypes based on right heart catheterization (RHC): precapillary, postcapillary, combined pre and postcapillary, and unclassified PH [6]. Precapillary PH is characterized by elevated pulmonary vascular resistance (PVR), reflecting intrinsic pulmonary vascular remodeling as seen in PoPH. Postcapillary PH is characterized by increased pulmonary artery wedge pressure (PAWP), typically resulting from volume overload or left heart dysfunction. Combined PH shows features of both elevated PVR and elevated PAWP, whereas unclassified PH refers to cases that do not meet strict criteria for either category. Differentiating these phenotypes is essential, as they reflect distinct pathophysiological mechanisms and may have different implications for perioperative management and prognosis.

Several studies have examined the prognostic significance of PH in LT; however, all have used the previous hemodynamic definition of PH, requiring a mean pulmonary artery pressure (mPAP) ≥ 25 mmHg. For example, recent reports have evaluated outcomes in patients with postcapillary PH [7], those with elevated mPAP ≥ 35 mmHg at transplantation [8], and cohorts comparing PoPH with pulmonary venous hypertension [9]. Although these investigations provided important insights into the heterogeneous hemodynamic phenotypes in cirrhosis, none incorporated the updated 6th World Symposium definition and the 2022 European Society of Cardiology/European Respiratory Society guidelines, which lowered the diagnostic threshold to mPAP > 20 mmHg and PVR ≥ 2 Wood units (WU) [6]. To date, there are no studies that have applied this new definition to comprehensively characterize PH subtypes—including precapillary, postcapillary, combined, and unclassified phenotypes—in the LT setting. In addition, PH is well known to negatively impact morbidity and mortality in patients undergoing cardiac [10] and noncardiac surgery [11]. It has specifically been associated with perioperative major adverse cardiovascular events [12], of which there is also limited investigation in the LT field.

Therefore, the aim of the present study is to investigate the association between ESLD patients with different etiologies of PH, based off newly updated hemodynamic classifications, and adverse cardiac events (ACEs) after LT. By comprehensively classifying patients into four PH phenotypes, we sought to clarify their respective prognostic implications in the context of LT. This approach may contribute to the growing body of evidence supporting risk assessment and perioperative decision‐making in patients with PH undergoing LT.

## 2. Materials and Methods

This was a single center retrospective observational study. All patients aged 18 years or more who underwent either living donor or deceased donor liver transplant between 2004 and 2023 were included in this study. This study was approved by the institutional review board (STUDY20050148) and conducted in accordance with the Declaration of Helsinki. The requirement for written informed consent was waived by the institutional review board owing to the retrospective design of the study. This study adheres to the Strengthening the Reporting of Observational Studies in Epidemiology (STROBE) [13] statement of reporting observational studies.

### 2.1. Study Population

**Figure 1:**
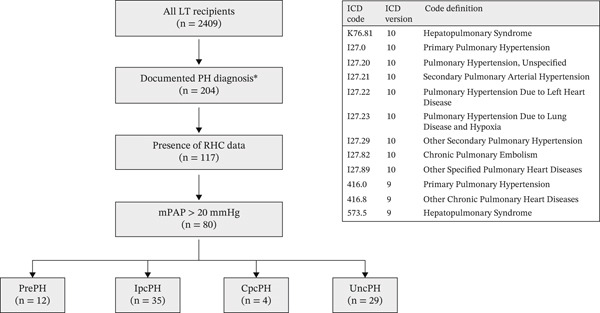
Patient selection and classification by pulmonary hemodynamic profile. Abbreviations: CpcPH, combined pre and postcapillary; ICD, International Classification of Diseases; IpcPH, isolated postcapillary; LT, liver transplantation; mPAP, mean pulmonary artery pressure; PH, pulmonary hypertension; PrePH, precapillary; RHC, right heart catheterization; UncPH, unclassified.

A step‐wise approach was used to determine the population of patients undergoing LT with confirmed preexisting PH (Figure 1). Patients were initially screened through the electronic medical record for a diagnosis of PH at the time of presentation for surgery using the International Classification of Diseases (ICD) Codes and corresponding code definitions (Figure 1). Given reports that PoPH and hepatopulmonary syndrome can coexist [14], we included hepatopulmonary syndrome in the initial screen. Patients with positive screening were then individually investigated for the presence of a RHC report prior to the date of LT. Patients with mPAP > 20 mmHg on RHC were included in the study. Demographic, clinical, and hemodynamic variables were manually extracted for analysis. Patients undergoing simultaneous liver–heart or liver–lung transplantation were excluded from the study. Patients undergoing simultaneous liver‐kidney transplantation were included. Patients undergoing repeat LT were included if the repeat liver transplant was > 30 days from the initial liver transplant.

### 2.2. Patient Classification

Patients were classified by RHC hemodynamic parameters according to the 2022 European Society of Cardiology/European Respiratory Society guidelines [6] as follows: (1) precapillary PH (PrePH): PAWP ≤ 15 mmHg and PVR > 2 WU, (2) isolated postcapillary PH (IpcPH): PAWP > 15 mmHg and PVR ≤ 2 WU, (3) combined pre and postcapillary PH (CpcPH): PAWP > 15 mmHg and PVR > 2 WU, or (4) unclassified PH (UncPH): PAWP < 15 mmHg and PVR ≤ 2 WU. If patients had multiple RHC prior to LT, the most recent RHC data was used for classification.

### 2.3. Outcomes

The primary outcome was a composite outcome of ACEs within 30 days of transplantation, inclusive of intraoperative events, and was defined as any of the following: cardiac arrest, acute coronary syndrome, stroke, right ventricular failure, new onset or exacerbation of congestive heart failure, and new onset or unstable arrhythmia (e.g., atrial fibrillation requiring pharmacologic or electrical cardioversion). Secondary outcomes including postoperative acute kidney injury (AKI), prolonged postoperative intubation (defined as > 24 h postoperatively), hospital length of stay after transplantation, in‐hospital mortality, and mortality at 30 days, 6 months, and 1 year posttransplantation. AKI was defined by the Kidney Disease Improving Global Outcomes (KDIGO) [15] criteria, and those on hemodialysis preoperatively were excluded from this outcome.

### 2.4. Statistical Analysis

Data are described using mean and standard deviation for normally distributed continuous variables, median and quartiles for nonnormally distributed continuous variables, and frequency (%) for categorical variables. Shapiro–Wilk test was used to determine the distribution of continuous variables. Baseline characteristics were compared among groups using the Kruskal–Wallis test for continuous variables and the Fisher′s exact test for categorical variables. Univariable logistic regression analyses were conducted to identify potential risk factors associated with the primary outcome. Variables included in the multivariable logistic regression model were selected based on their clinical relevance and prior literature, as well as consideration of the limited number of outcome events. Age and MELD score were included as key clinical factors commonly reported in previous studies [7], whereas mPAP and PAWP were chosen as representative hemodynamic parameters. PVR was excluded because it is derived from PAWP and other measures. Results are expressed as odds ratios (ORs) with 95% confidence intervals (CIs). Kaplan–Meier curves were generated to evaluate the cumulative incidence of the primary outcome, and survival curves were compared using the log‐rank test. A two‐sided *p* value < 0.05 was considered statistically significant. Statistical analyses were performed using R Version 4.4.3.

## 3. Results

### 3.1. Patients

Over the study period, 2409 patients underwent LT, and 80 patients (3.3%) met criteria for inclusion. The distribution of patients in each group were as follows: 12 for PrePH, 35 for IpcPH, 4 for Cpc PH, and 29 for UncPH. There were no statistically significant differences in baseline characteristics (Table 1) between the groups including age, sex, history of hypertension, diabetes, renal failure on hemodialysis, MELD score, etiology of cirrhosis, presence of hepatocellular carcinoma, or use of a living liver donor.

**Table 1:** Patient characteristics.

	PrePH (*n* = 12)	IpcPH (*n* = 35)	CpcPH (*n* = 4)	UncPH (*n* = 29)	*p*
Age, years	57.5 (51, 66.5)	58 (51, 64)	53.5 (41.5, 58.5)	60 (53, 66)	0.46
Sex, male, *n* (%)	6 (50%)	20 (57.1%)	0 (0.0%)	13 (44.8%)	0.19
Hypertension, *n* (%)	2 (16.7%)	18 (51.4%)	1 (25.0%)	9 (31.0%)	0.12
Diabetes, *n* (%)	6 (50.0%)	11 (31.4%)	2 (50.0%)	6 (20.7%)	0.21
Dialysis, *n* (%)	1 (8.3%)	4 (11.4%)	1 (25.0%)	3 (10.3%)	0.74
MELD Score	22 (19, 26.5)	23 (18, 29)	32 (29, 36.5)	22 (15, 27)	0.08
Simultaneous kidney, *n* (%)	1 (8.3%)	2 (5.7%)	0 (0.0%)	0 (0.0%)	0.47
Repeat transplant, *n* (%)	0 (0.0%)	2 (5.7%)	0 (0.0%)	1 (3.5%)	0.80
Living donor, *n* (%)	3 (25.0%)	12 (34.3%)	0 (0.0%)	12 (41.4%)	0.43
HCC, *n* (%)	4 (33.3%)	2 (5.7%)	0 (0.0%)	4 (13.8%)	0.08
CAD, *n* (%)	0 (0%)	3 (8.6%)	0 (0%)	3 (10.3%)	0.64
Arrhythmia, *n* (%)	2 (16.7%)	6 (17.1%)	1 (25.0%)	5 (17.2%)	0.98
CHF, *n* (%)	1 (8.3%)	2 (5.7%)	0 (0%)	1 (3.5%)	0.88
Valvular disease, *n* (%)	1 (8.3%)	2 (5.7%)	0 (0%)	4 (13.8%)	0.63
CVA, *n* (%)	0 (0%)	0 (0%)	0 (0%)	0 (0%)	—
Thromboembolism, *n* (%)	1 (8.3%)	2 (5.7%)	0 (0%)	0 (0%)	0.50
Etiology					0.39
Alcoholic, *n* (%)	3 (25.0%)	11 (31.4%)	1 (25.0%)	11 (37.9%)	
NASH, *n* (%)	1 (8.3%)	9 (25.7%)	1 (25.0%)	9 (31.0%)	
HCV, *n* (%)	3 (25.0%)	5 (14.3%)	0 (0.0%)	5 (17.2%)	
PBC, *n* (%)	3 (25.0%)	1 (2.9%)	1 (25.0%)	1 (3.5%)	
PSC, *n* (%)	0 (0.0%)	1 (2.9%)	0 (0.0%)	1 (3.5%)	
Multiple, *n* (%)	0 (0.0%)	2 (5.7%)	1 (25.0%)	1 (3.5%)	
Idiopathic/cryptogenic, *n* (%)	1 (8.3%)	4 (11.4%)	0 (0.0%)	1 (3.5%)	
Other, *n* (%)	0 (0.0%)	2 (5.7%)	(0.0%)	0 (0.0%)	
LVEF, %	57.5 (55, 60)	60 (55, 65)	57.5 (55, 65)	60 (55, 65)	0.37
mPAP, mmHg	29 (23.5, 31)	28 (23, 32)	38 (33.5, 43)	24 (22, 26)	0.0001
PAWP, mmHg	9.5 (8.5, 11.5)	18 (17, 23)	19.1 (18.1, 20.9)	12 (11, 14)	0.0001
PVR, WU	2.5 (2.4, 2.7)	0.83 (0.62, 1.3)	2.9 (2.5, 4.3)	1.1 (0.95, 1.5)	0.0001
CO, L/min	7.3 (6.1, 8.6)	8.5 (6.7, 10.6)	6.4 (4.9, 7.3)	9.1 (7.5, 11.7)	0.01
RAP, mmHg	4.5 (2, 6)	11 (8, 14)	18.5 (9, 20.2)	6 (5, 11)	0.0001

Abbreviations: CAD, coronary artery disease; CHF, congestive heart failure; CO, cardiac output; CVA, cerebrovascular accident; HCC, hepatocellular carcinoma; HCV, hepatitis C virus; LVEF, left ventricular ejection fraction; MELD, model for end‐stage liver disease; mPAP, mean pulmonary artery pressure; NASH, nonalcoholic steatohepatitis; PAWP, pulmonary artery wedge pressure; PBC, primary biliary cholangitis; PH, pulmonary hypertension; PSC, primary sclerosing cholangitis; PVR, pulmonary vascular resistance; RAP, right atrial pressure.

### 3.2. Hemodynamic Characteristics

The hemodynamic parameters of each group classification are shown in Table 1. The average mPAP was highest in the CpcPH group (38 [33.5–43] mmHg), followed by the PrePH group (29 [23.5–31] mmHg), IpcPH group (28 [23–32] mmHg), and UncPH group (24 [22–26] mmHg). Appropriately, PVR was elevated in the PrePH and CpcPH groups, and PAWP was elevated in the IpcPH and CpcPH groups. Cardiac output (CO) was comparatively lower in the CpcPH group.

### 3.3. Primary Outcome

**Figure 2:**
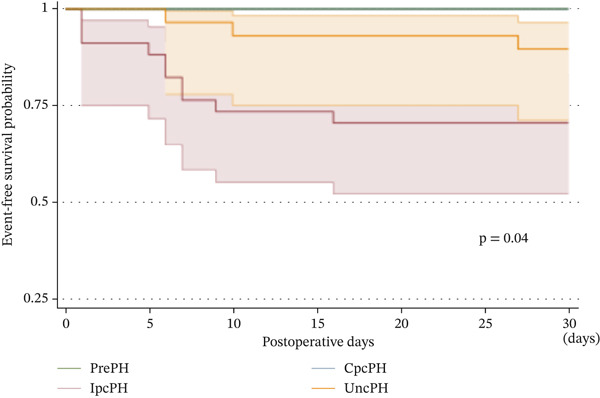
Kaplan–Meier estimates of 30‐day ACE‐free survival by pulmonary hypertension classification. Abbreviations: CpcPH, combined pre and postcapillary; IpcPH, isolated postcapillary; PrePH, precapillary; UncPH, unclassified.

ACEs occurred in 15 of the 80 patients, including 11 patients in the IpcPH group, 1 patient in the CpcPH group, and 3 patients in the UncPH group, whereas no events occurred in the PrePH group (Table 2). The incidences of ACEs were 0%, 31.4%, 25.0%, and 10.3% (*p* = 0.04) for the PrePH, IpcPH, CpcPH, and UncPH groups, respectively. Cumulative incidences of ACEs by groups are shown in Figure 2. Of all causes of ACEs, arrhythmia was the most frequent (*n* = 9), followed by cardiac arrest (*n* = 3) and congestive heart failure (*n* = 3). Other causes included death (*n* = 1) and stroke (*n* = 1).

**Table 2:** Primary and secondary outcomes.

	PrepH (*n* = 12)	IpcPH (*n* = 35)	CpcPH (*n* = 4)	UncPH (*n* = 29)	*p*
Adverse cardiac events, *n* (%)	0 (0.0%)	11 (31.4%)	1 (25.0%)	3 (10.3%)	0.04
AKI, *n* (%)	6 (50.0%)	21 (60.0%)	1 (25.0%)	16 (55.2%)	0.81
Prolonged intubation, *n* (%)	6 (50.0%)	15 (42.9%)	4 (100.0%)	16 (55.2%)	0.19
LOS, days	15.5 (11, 38.5)	15 (8, 34)	21.5 (11.5, 36)	16 (10, 25)	0.89
In‐hospital mortality, *n* (%)	0 (0.0%)	3 (8.6%)	0 (0.0%)	0 (0.0%)	0.38
Mortality at 30 days, *n* (%)	0 (0.0%)	1 (2.9%)	0 (0.0%)	0 (0.0%)	0.73
Mortality at 6 months, *n* (%)	0 (0.0%)	3 (8.6%)	0 (0.0%)	3 (10.3%)	0.81
Mortality at 1 year, *n* (%)	0 (0.0%)	3 (8.6%)	0 (0.0%)	5 (17.2%)	0.48

Abbreviations: AKI, acute kidney injury; LOS, length of stay.

Baseline characteristics were compared between patients with and without ACEs (Table 3). Patients with ACEs had significantly higher PAWP (18.1 [16–23] vs. 14 [12–18] mmHg, *p* = 0.01) and lower PVR (0.79 [0.50–1.4] vs. 1.3 [0.86–1.9] WU, *p* = 0.01) compared with those without ACEs, while mPAP and CO were similar between groups. Other baseline variables, including age, sex, comorbidities, MELD score, donor type, and etiology, also did not differ significantly between groups. In a multivariable logistic regression analysis (Table 4) including age, MELD score, mPAP, and PAWP, only PAWP was independently associated with ACEs (OR, 1.19 [95% CI, 1.04–1.36]; *p* = 0.01).

**Table 3:** Comparison of variables by outcome status.

Variables	Without ACEs (*n* = 65)	With ACEs (*n* = 15)	*p*
Age, years	59 (51, 65)	59 (52, 64)	0.73
Sex, male, *n* (%)	31 (47.7%)	8 (53.3%)	0.78
Hypertension, *n* (%)	24 (36.9%)	6 (40.0%)	1.00
Diabetes, *n* (%)	20 (30.8%)	5 (33.3%)	1.00
Dialysis, *n* (%)	7 (10.8%)	2 (13.3%)	0.67
PH Type			0.04
PrePH, *n* (%)	12 (18.5%)	0 (0.0%)	
IpcPH, *n* (%)	24 (36.9%)	11 (73.3%)	
CpcPH, *n* (%)	3 (4.6%)	1 (6.7%)	
UncPH, *n* (%)	26 (40.0%)	3 (20.0%)	
MELD score	22 (17, 29)	24 (18, 27)	0.98
Donor, living, *n* (%)	22 (33.9%)	5 (33.3%)	1.00
Simultaneous kidney, *n* (%)	2 (3.1%)	1 (6.7%)	0.47
Repeat transplant, *n* (%)	1 (1.5%)	2 (13.3%)	0.09
HCC, *n* (%)	9 (13.9%)	1 (6.7%)	0.68
CAD, *n* (%)	4 (6.2%)	2 (13.3%)	0.34
Arrhythmia, *n* (%)	9 (13.9%)	5 (33.3%)	0.07
CHF, *n* (%)	3 (4.6%)	1 (6.7%)	0.74
Valvular disease, *n* (%)	5 (7.7%)	2 (13.3%)	0.49
CVA, *n* (%)	0 (0%)	0 (0%)	—
Thromboembolism, *n* (%)	3 (4.6%)	0 (0%)	0.40
Etiology			0.19
Alcoholic, *n* (%)	22 (33.9%)	4 (26.7%)	
NASH, *n* (%)	17 (26.2%)	3 (20.0%)	
HCV, *n* (%)	11 (16.9%)	2 (13.3%)	
PBC, *n* (%)	5 (7.7%)	1 (6.7%)	
PSC, *n* (%)	2 (3.1%)	0 (0.0%)	
Multiple, *n* (%)	4 (6.2%)	0 (0.0%)	
Idiopathic/cryptogenic, *n* (%)	2 (3.1%)	4 (26.7%)	
Other, *n* (%)	1 (1.5%)	1 (6.7%)	
LVEF, %	60 (55, 65)	60 (55, 65)	0.71
MPAP, mmHg	25 (23, 30)	28 (23, 30)	0.55
PAWP, mmHg	14 (12, 18)	18.1 (16, 23)	0.01
PVR, WU	1.3 (0.86, 1.9)	0.79 (0.50, 1.4)	0.01
CO, L/min	8.3 (6.9, 10.3)	9.1 (7.2, 11.7)	0.36
RAP, mmHg	7 (5, 10)	10 (6, 15)	0.06

Abbreviations: ACE, adverse cardiac events; CAD, coronary artery disease; CHF, congestive heart failure; CO, cardiac output; CVA, cerebrovascular accident; HCC, hepatocellular carcinoma; HCV, hepatitis C virus; LVEF, left ventricular ejection fraction; MELD, model for end‐stage liver disease; mPAP, mean pulmonary artery pressure; NASH, nonalcoholic steatohepatitis; PAWP, pulmonary artery wedge pressure; PBC, primary biliary cholangitis; PH, pulmonary hypertension; PSC, primary sclerosing cholangitis; PVR, pulmonary vascular resistance; RAP, right atrial pressure.

**Table 4:** Multivariable analysis of risk factors for adverse cardiac events.

Variable	OR	95% CI	*p*
Age, years	1.02	0.94–1.11	0.61
MELD	0.99	0.90–1.08	0.76
mPAP, mmHg	0.93	0.79–1.08	0.34
PAWP, mmHg	1.19	1.04–1.36	0.01

Abbreviations: MELD, model for end‐stage liver disease; mPAP, mean pulmonary artery pressure; PAWP, pulmonary artery wedge pressure.

### 3.4. Secondary Outcomes

There were no significant differences in the rates of AKI (*p* = 0.81), prolonged intubation (*p* = 0.19), or hospital length of stay (*p* = 0.89). Mortality during hospitalization, at 30 days, 6 months, and 1 year was also similar among the groups (*p* = 0.38, *p* = 0.73, *p* = 0.81, and *p* = 0.48, respectively).

## 4. Discussion

This study showed that, among patients with mild to low moderate PH confirmed by RHC, PAWP, and not PVR, was associated with ACEs after LT. Patients with IpcPH, characterized by elevated PAWP and normal PVR—that is, patients in whom PH was secondary to heart disease—had the highest incidence of postoperative ACEs. The PrePH group did not have any postoperative ACEs, and patients with precapillary PH who did have ACEs also concurrently had postcapillary PH.

PoPH is regarded as a relative contraindication to LT. Krowka et al. [16] found 100% mortality in patients with severe PoPH and 50% mortality in patients with moderate PoPH, leading to the suggestion that mPAP should be optimized to be below 35 mmHg prior to LT . In fact, MELD exception is granted to patients with PoPH if posttreatment mPAP can be maintained less than 35 mmHg [17]. However, these recommendations have been generalized to all ESLD patients with PH, even those with postcapillary etiology.

To our knowledge, few studies have directly compared posttransplant outcomes across different PH phenotypes in liver transplant candidates. Rajaram et al. [9] conducted a single‐center retrospective study that stratified patients into no PH, PoPH, and pulmonary venous hypertension groups, where the pulmonary venous hypertension group is equivalent to our IpcPH group. They reported that patients with PoPH had lower one‐year survival compared to those with pulmonary venous hypertension or without PH at all, whereas there were no significant differences in short‐term mortality or other perioperative outcomes such as AKI, ventilator days, or intensive care unit length of stay. In contrast, our study expands on these findings in several important ways. First, we analyzed RHC data obtained at the time closest to transplantation, rather than at the initial evaluation, which may better reflect the hemodynamic status immediately preceding surgery. Second, we included both combined pre and postcapillary PH as well as unclassified PH groups, providing a broader representation of the heterogeneity frequently encountered in clinical practice. Third, we applied the updated definition of PH with a mPAP threshold of > 20 mmHg, allowing us to detect clinically meaningful associations even in patients with milder disease. Although we did not observe significant differences in mortality, we identified an increased incidence of perioperative ACEs among patients with postcapillary pathophysiology. Although ACEs have been linked to higher long‐term mortality in other surgical populations [18], our sample size may have limited the ability to detect such associations in the liver transplant setting. Nevertheless, given the paucity of prior studies directly comparing PH phenotypes in the liver transplant population, our analysis suggests the importance of distinguishing precapillary from postcapillary physiology when evaluating candidates.

This study has several limitations. First, it was a retrospective single‐center analysis, which may limit the generalizability of our findings. Second, the sample size of this study was constrained by the availability of RHC data, reducing statistical power to detect differences in less frequent outcomes such as mortality. In addition, because the initial screening was based on ICD code diagnosis, patients that may have qualified for PH diagnosis based off new guidelines were not included. Furthermore, because there was exclusion of a large proportion of patients with a PH diagnosis due to lack of RHC data, a selection bias that introduces an unknown confounder cannot be ruled out. Third, we did not incorporate echocardiographic measures such as right ventricular strain or dilation and left ventricular diastolic dysfunction, which could have provided additional insight into the mechanisms underlying ACEs. Fourth, outcome ascertainment was confined to perioperative and 1‐year follow‐up, and therefore longer‐term prognostic implications of PH phenotypes could not be evaluated. Finally, unmeasured confounders, including donor‐related factors such as graft quality or donation type and intraoperative management strategies such as vasopressor use, transfusion practices, or venovenous bypass, were not accounted for and may have influenced postoperative outcomes.

In conclusion, in patients with mild to low moderate PH confirmed by RHC presenting for LT, it is important to characterize the hemodynamic profile associated with the PH etiology. It is certainly necessary for treatment planning, but it may also have prognostic implications and play a role in risk stratification. Although this study does not advocate for performing RHC specifically to ascertain hemodynamic details, it does provide insight into the interpretation of existing RHC data, especially in the context of newly established definitions of PH. Future large‐scale, prospective studies are warranted to validate these findings and overcome the limitations of the present analysis.

## Funding

No funding was received for this manuscript.

## Conflicts of Interest

The authors declare no conflicts of interest.

## Data Availability

The data that support the findings of this study are available on request from the corresponding author. The data are not publicly available due to privacy or ethical restrictions.
